# *Pseudomonas aeruginosa* polysaccharide Psl supports airway microbial community development

**DOI:** 10.1038/s41396-022-01221-y

**Published:** 2022-03-25

**Authors:** Sara N. Stoner, Joshua J. Baty, Jessica A. Scoffield

**Affiliations:** Department of Microbiology, University of Alabama at Birmingham, Birmingham, AL USA

**Keywords:** Bacteriology, Biofilms, Microbiome, Clinical microbiology

## Abstract

*Pseudomonas aeruginosa* dominates the complex polymicrobial cystic fibrosis (CF) airway and is a leading cause of death in persons with CF. Oral streptococcal colonization has been associated with stable CF lung function. However, no studies have demonstrated how *Streptococcus salivarius*, the most abundant streptococcal species found in individuals with stable CF lung disease, potentially improves lung function or becomes incorporated into the CF airway biofilm. By utilizing a two-species biofilm model to probe interactions between *S. salivarius* and *P. aeruginosa*, we discovered that the *P. aeruginosa* exopolysaccharide Psl promoted *S. salivarius* biofilm formation. Further, we identified a *S. salivarius* maltose-binding protein (MalE) that is required for promotion of biofilm formation both in vitro and in a *Drosophila melanogaster* co-infection model. Finally, we demonstrate that promotion of dual biofilm formation with *S. salivarius* is common among environmental and clinical *P. aeruginosa* isolates. Overall, our data supports a model in which *S. salivarius* uses a sugar-binding protein to interact with *P. aeruginosa* exopolysaccharide, which may be a strategy by which *S. salivarius* establishes itself within the CF airway microbial community.

## Introduction

Cystic fibrosis (CF) is the most common lethal genetic disorder in Caucasian populations [1]. Individuals with this disorder accumulate thick mucus in the lungs, and the inability to clear this mucus from the airways facilitates the colonization of microbes [1]. The bacterial species *Pseudomonas aeruginosa* is the leading cause of death for individuals with CF [2]. The prevalence of *P. aeruginosa* colonization in the CF population increases with age, with up to 60% of CF adults being colonized in their lifetime [3]. *P. aeruginosa* uses various strategies to cause persistent infections in the lung, including evasion of the host immune system, conversion to a mucoid phenotype, and biofilm formation [2]. These adaptation mechanisms, particularly the ability to form recalcitrant biofilms, render *P. aeruginosa* difficult to treat with antibiotics and permit the development of lifelong chronic infections that lead to rapid lung deterioration and mortality [4].

Biofilms are defined as a community of microbes that are attached to a surface and embedded in a protective extracellular matrix [5]. *P. aeruginosa* produces multiple exopolysaccharides that comprise its biofilm matrix. Non-mucoid strains *of P. aeruginosa*, which typically colonize the CF lung initially, produce the exopolysaccharides Pel and Psl [6]. Over the course of infection, non-mucoid strains will accumulate mutations and convert to a mucoid phenotype by switching to production of the exopolysaccharide alginate [7]. Pel, Psl, and alginate play an important role in antimicrobial resistance by preventing penetration of antibiotics into the *P. aeruginosa* biofilm [8, 9]. Additionally, Psl is important for *P. aeruginosa* integration into polymicrobial biofilms [10].

Only in recent years have researchers began to study how cross-species interactions in biofilms influence the composition of polymicrobial communities [10]. The importance of studying *P. aeruginosa* interactions with other microbes in the CF airway polymicrobial community is becoming increasingly recognized [11, 12]. For instance, *P. aeruginosa* has been found to synthesize glutamate from precursor molecules secreted by *Rothia mucilaginosa*, another common microbe found in the CF lung [13]. Additionally, Psl produced by *P. aeruginosa* has been shown to interact with the staphylococcal protein A of *Staphylococcus aureus* to increase *P. aeruginosa* resistance to antibiotics [14]. Lastly, colonization of *Stenotrophomonas maltophilia*, an emerging CF pathogen, is promoted in murine lungs through integration into *P. aeruginosa* biofilms [15]. Relevant to our study, oral streptococci are increasingly recognized as core residents of the CF lung microbiota. Historically, these bacterial taxa have been thought to reside solely in the oral cavity and any detection of these microbes outside of this environment, particularly in the CF lung, was thought to be transient or attributed to oral contamination during sample collection. Multiple independent microbiome studies using sputum and bronchoalveolar lavage fluid have confirmed the presence of mitis and salivarius group oral streptococci in the CF airway [16–19]. Oral commensal streptococci have been shown to be associated with lung stability and increased microbial diversity in CF individuals. *Streptococcus salivarius* is the most prevalent streptococcal species found in the lungs of individuals with stable CF lung disease [16]. We previously reported that the second most abundant oral commensal found in the CF airway, *Streptococcus parasanguinis*, adheres to the mucoid *P. aeruginosa* exopolysaccharide alginate, resulting in the promotion of *S. parasanguinis* biofilm formation [20]. Therefore, it is important to understand how these commensals are incorporated into the CF polymicrobial community and potentially impact lung function in the CF population.

*S. salivarius* has been shown to commonly colonize the upper respiratory tract of infant children [21, 22]. Because many individuals with CF become colonized with *P. aeruginosa* during adulthood, it is likely that *S. salivarius* is present in the lungs during the early stages of infection with non-mucoid *P. aeruginosa* [3]. In an effort to better understand how *S. salivarius* incorporates into biofilms with the major CF pathogen *P. aeruginosa*, we utilized two species in vitro and in vivo biofilm models to identify mechanisms that facilitate *S. salivarius* colonization. Here, we report that *S. salivarius* exploits the non-mucoid *P. aeruginosa* exopolysaccharide Psl to promote streptococcal biofilm formation. This enhanced biofilm phenotype was consistent when *P. aeruginosa* environmental and clinical isolates were grown with *S. salivarius*. Moreover, we found that the presence of a streptococcal maltose-binding surface protein, MalE, potentially facilitates the interaction between *S. salivarius* and *P. aeruginosa* Psl. Finally, we show that *P. aeruginosa* promotes *S. salivarius* colonization in a *Drosophila melanogaster* in vivo model of co-infection. Taken together, our study highlights a unique mechanism by which *S. salivarius* utilizes *P. aeruginosa* extracellular components to influence the CF airway microbial community by initiating and sustaining streptococcal colonization within the CF lung.

## Results

### Non-mucoid *Pseudomonas aeruginosa* promotes *Streptococcus salivarius* biofilm formation

**Fig. 1:**
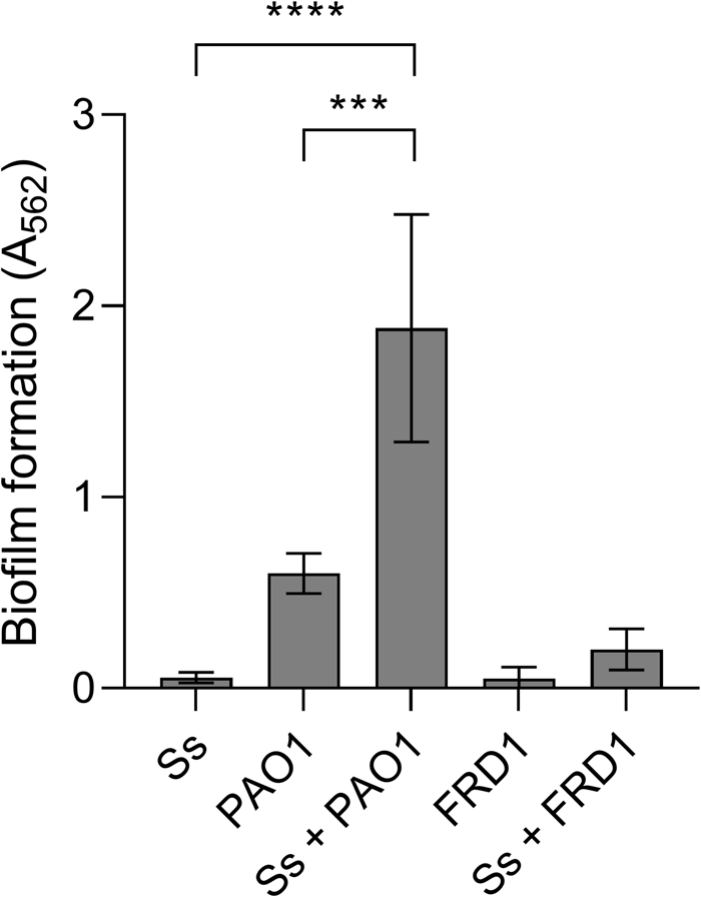
*S. salivarius* and *P. aeruginosa* produce an enhanced biofilm in a dual species model. *S. salivarius* (Ss) was co-cultured with either of two *P. aeruginosa* strains (PAO1 and FRD1) in TSBYE medium in a 96-well plate for 16 h at 37 °C (*n* = 3 biological replicates, 6 technical). Biofilm biomass was then measured using crystal violet staining. One-way ANOVA with Šίdák’s multiple comparisons test. Error bars indicate standard deviation. ***p < 0.001, ****p < 0.0001.

**Fig. 2:**
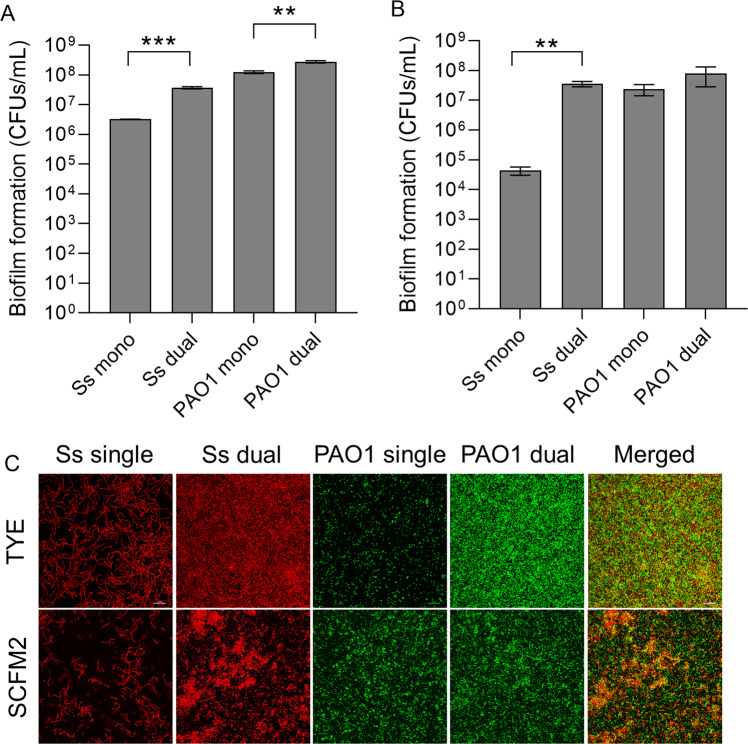
Non-mucoid *P. aeruginosa* strain PAO1 promotes *S. salivarius* biofilm formation. **A** Quantification of Ss and PAO1 biofilm CFUs was performed in a 6-h, 6-well mono- and dual-species biofilm model. Samples were cultured in TSBYE medium and **B** synthetic CF sputum (SCFM2), serially diluted and plated on THB agar. Data represent three biological replicates performed in triplicate. Student’s *t* test. Error bars indicate mean ± SD. **C** Confocal microscopy was performed on Ss and PAO1-GFP single and dual species biofilms in both TSBYE and SCFM2. Ss was stained with hexidium iodide. **p < 0.01, ***p < 0.001.

To characterize interactions between the oral commensal *S. salivarius* and the CF lung pathogen *P. aeruginosa*, we co-cultured *S. salivarius* with an acute wound isolate (PAO1) and chronic CF isolate (FRD1). *S. salivarius* and the acute isolate PAO1 formed significantly more biofilm biomass when co-cultured compared to single species controls (Fig. 1). When we co-cultured *S. salivarius* with the mucoid isolate FRD1, biofilm formation did not increase compared to the single species controls (Fig. 1). To examine the relative species contribution in dual species biofilms with *S. salivarius* and PAO1, both planktonic and biofilm colony forming units (CFUs) were enumerated (Fig. 2A). When co-cultured in TSBYE, both *S. salivarius* and *P. aeruginosa* biofilm CFUs increased significantly compared to single species controls. In contrast, no change in planktonic cell number was observed for either *S. salivarius* or *P. aeruginosa* in the presence of the other species (Fig S1A). We also co-cultured *S. salivarius* and *P. aeruginosa* in a synthetic cystic fibrosis sputum medium known as SCFM2, which mimics the nutrient profile found in the sputum of persons with CF [23]. When cultured in SCFM2, we observed an even greater increase in *S. salivarius* biofilm CFUs in the presence of *P. aeruginosa* (Fig. 2B). However, *P. aeruginosa* planktonic and biofilm cell number did not increase in the presence of *S. salivarius* in SCFM2, suggesting that this interspecies interaction exclusively enhances *S. salivarius* biofilm growth in synthetic CF sputum (Fig. 2 (Fig S1B)), which better recapitulates the nutritional environment in the CF airway [23]. We confirmed these observations via confocal laser microscopy and observed an increase in *S. salivarius* and *P. aeruginosa* biofilm formation in TSBYE when co-cultured, and an increase only in *S. salivarius* biofilm formation in SCFM2 (Fig. 2C). Additionally, propidium iodide staining, which stains dead cells or nucleic acids [24, 25] was prominent in our dual biofilm TSBYE group (Fig S2). We did not observe a reduction in planktonic cells for either species in the dual biofilm group, suggesting that this staining is likely due to extracellular DNA, an important component of the biofilm matrix [24, 26]. Enhanced *S. salivarius* biofilm formation in the presence of *P. aeruginosa* strain PAO1, but not FRD1 suggests that PAO1 possesses a distinct feature that promotes *S. salivarius* biofilm formation.

### *P. aeruginosa* exopolysaccharide Psl enhances *S. salivarius* biofilm formation

**Fig. 3:**
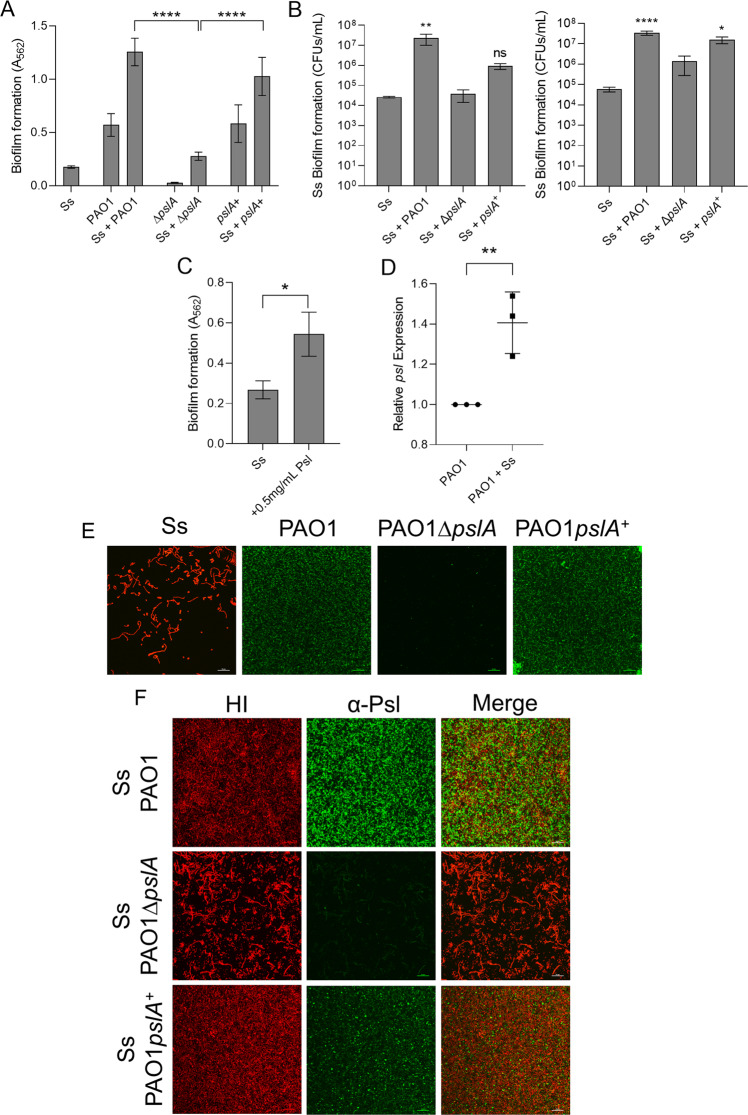
*P. aeruginosa* exopolysaccharide Psl promotes *S. salivarius* biofilm formation. Ss was co-cultured with *P. aeruginosa* PAO1 strains (**A**) PAO1∆*pslA* and PAO1 *pslA*^*+*^ in TSBYE medium with 1% sucrose in a 96-well plate for 16 h at 37 °C with 5% CO_2_ (*n* = 3 biological replicates, 3 technical). Biofilm biomass was then measured using crystal violet staining. One-way ANOVA with Dunnett’s multiple comparisons test. **B** Quantification of Ss biofilm-forming cells after co-culturing with PAO1, PAO1∆*pslA*, and PAO1 *pslA*^*+*^ in TSBYE (left) and SCFM2 [50] in a 6-h, 6-well model at 37 °C with 5% CO_2_ (*n* = 3 biological replicates, each with 3 technical replicates). One-way ANOVA with Šίdák’s multiple comparisons test. **C** 0.5 mg/mL purified Psl was added to Ss single cultures in TSBYE with 1% sucrose in a 96-well 16-h biofilm. Crystal violet staining was used to quantify biofilm biomass. **D** qPCR quantification of *P. aeruginosa pslA* expression compared to 16S rRNA control. Student’s *t* test. Fluorescence microscopy images at 60× magnification of 16-h single (**E**) and dual species (**F**) biofilms of Ss and PAO1, PAO1*∆pslA*, and PAO1*pslA*^+^ cultured in TSBYE supplemented with 1% sucrose. Ss was stained with hexidium iodide, and Psl was stained with a FITC-conjugated α-Psl monoclonal antibody. Scale bar: 20 μm. *p < 0.05, **p < 0.01, ****p < 0.0001.

One major difference between the two strains is the exopolysaccharides produced within their respective biofilm matrices. Initial CF infecting *P. aeruginosa* strains that are acquired from the environment resemble PAO1 and produce Pel and Psl, whereas CF adapted strains like FRD1 overproduce alginate, largely due to accumulation of mutations in the *mucA* gene [6, 7]. To investigate whether production of the exopolysaccharide Psl, the most prominent exopolysaccharide made by PAO1, promotes *S. salivarius* biofilm formation, we co-cultured *S. salivarius* biofilms with a PAO1 mutant deficient in Psl (PAO1Δ*pslA*), and PAO1Δ*pslA* complemented with a wild-type copy of *pslA* (PAO1*pslA*^+^) [27]. When *S. salivarius* was co-cultured with PAO1*ΔpslA*, we observed significantly less dual biofilm formation compared to wild-type PAO1 co-cultures with *S. salivarius*. Co-culturing of *S. salivarius* with PAO1*pslA*^+^ restored the dual species biofilm to levels similar to the wild-type PAO1 dual biofilm (Fig. 3A). When quantifying *S. salivarius* biofilm CFUs in both TSBYE and SCFM2, we found that co-culture of *S. salivarius* with PAO1*ΔpslA* resulted in a significantly decreased *S. salivarius* biofilm cell number compared to *S. salivarius* co-cultured with wild-type PAO1 (Fig. 3B). When co-cultured with PAO1*pslA*^*+*^*, S. salivarius* biofilm was restored to levels similar to wild-type PAO1 in SCFM2 and partially restored in TSBYE, further confirming that Psl enhances *S. salivarius* biofilm formation. We quantified and compared planktonic CFUs of PAO1*ΔpslA* and PAO1*pslA*^*+*^ to wildtype PAO1 planktonic CFUs to confirm the lack of *S. salivarius* biofilm formation in the presence of *PAO1ΔpslA* was not caused by growth defects and lower cell density in the mutant strain co-culture (Figs. S3, S4). Additionally, we saw no growth advantage over time when we performed 16-h growth curves of wild-type PAO1 cultured with and without *S. salivarius*, further confirming that *S. salivarius* biofilm promotion is not due to increased growth of *P. aeruginosa* (Fig. S5). To further confirm that *P. aeruginosa* Psl does indeed contribute to enhanced biofilm formation by *S. salivarius*, we co-cultured *S. salivarius* with two *P. aeruginosa* isolates known to produce little to no Psl and measured changes in biofilm biomass [28, 29]. *P. aeruginosa* exopolysaccharide biosynthetic genes compete for the same precursor sugars, therefore, induction of one polysaccharide decreases the production of alternative exopolysaccharides [30]. Hence, overproduction of alginate in the *mucA* deficient strain of PAO1 results in a reduction in Psl production [29, 30]. Significantly less dual biofilm formation was observed with the PAO1*ΔmucA*-alginate overproducing strain in comparison to wild-type PAO1 (Fig. S6A). We observed a similar result when co-culturing *S. salivarius* with the *P. aeruginosa* strain PA14, which lacks essential Psl biosynthetic genes and solely produces the exopolysaccharide Pel (Fig. S6B) [31]. Additionally, purified Psl significantly increased the single species *S. salivarius* biofilm, further confirming the role of Psl in promoting *S. salivarius* (Fig. 3C). Lastly, *pslA*, a gene required for Psl production, was shown to be significantly upregulated in the presence of *S. salivarius* (Fig. 3D).

To understand the spatial relationship between *S. salivarius* and Psl within a dual biofilm, we performed confocal laser scanning microscopy on single and dual biofilms of *S. salivarius* and *P. aeruginosa* strains PAO1, PAO1*ΔpslA*, and PAO1*pslA*^+^ stained with a FITC-conjugated α-Psl antibody to further characterize the role of Psl in promotion of *S. salivarius* biofilm formation (Fig. 3E, F). Consistent with our biofilm CFU quantifications, we observed a significant increase in *S. salivarius* biofilm in the presence of wild-type PAO1 as well as PAO1*pslA*^+^. When co-cultured with PAO1*ΔpslA*, no increase in *S. salivarius* biofilm formation was observed. Both *S. salivarius* and Psl were dispersed throughout the wild-type PAO1 and PAO1*pslA*^+^ dual biofilms. Additionally, changes in *S. salivarius* biofilm architecture were observed in the presence of Psl (Fig. 3F). Overall, our findings show that Psl not only promotes *S. salivarius* biofilm development, but also modifies *S. salivarius* biofilm structure in dual species biofilms with *P. aeruginosa*.

To determine whether *S. salivarius* utilizes Psl only as a biofilm matrix scaffold or also metabolizes Psl, we tested whether *S. salivarius* could utilize purified Psl as a carbon source by monitoring planktonic growth in full-strength and 1:1 diluted THB media that was supplemented with Psl or glucose (Fig. S7). *S. salivarius* grew similarly in the presence of Psl compared to the no-sugar control in full strength THB. Conversely, *S. salivarius* growth was enhanced in the presence of purified Psl in diluted THB media compared to the no-sugar control. This finding suggests that *S. salivarius* metabolizes Psl under conditions in which preferred carbon sources are limited. Our results demonstrate that Psl promotes *S. salivarius* biofilm formation, as well as *S. salivarius* planktonic growth via metabolism of Psl under specific nutrient-limited conditions.

### *S. salivarius* maltose-binding protein MalE plays a role in promotion of *S. salivarius* biofilm formation both in vitro and in vivo

**Fig. 4:**
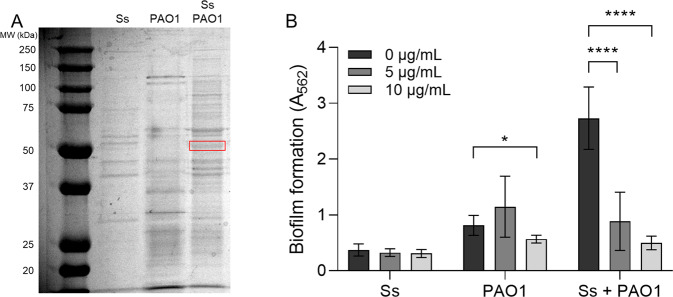
*S. salivarius* maltose-binding protein MalE promotes dual biofilm formation. **A** Ss and PAO1 were cultured individually and dually in TSBYE medium at 37 °C while shaking until OD_600_ = 1.8 was reached. Cells were resuspended in Tris-Buffered Saline (TBS) and lysed using the Bead Blaster 24 (Benchmark). Supernatant was run on SDS page gel, and overproduced bands in the dual sample were sent to the UAB Mass Spectrometry core for identification. **B** Ss and PAO1 were cultured in TSBYE with 1% sucrose in a 96-well 16-h biofilm model in the presence of 0 µg/mL, 5 µg/mL or 10 µg/mL α-MalE mAbs. Biofilms were stained with crystal violet to measure biofilm biomass (*n* = 3 biological, 3 technical). Error bars indicate mean ± SD. Two-way ANOVA with Tukey’s multiple comparisons test for post-hoc analysis. *p < 0.05, ****p < 0.0001.

**Fig. 5:**
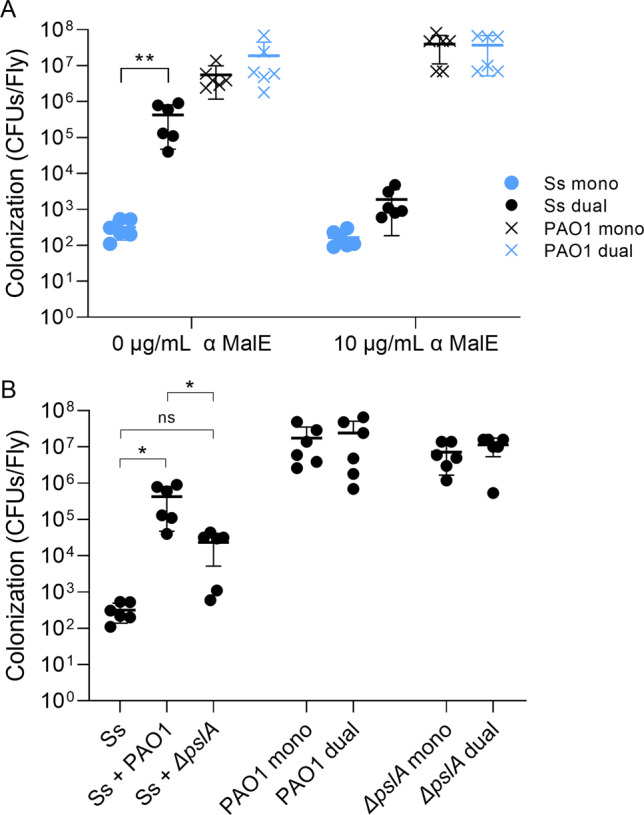
Promotion of *S. salivarius* colonization of *Drosophila* is MalE and Psl dependent. **A** After antibiotic treatment, *Drosophila* were infected with subcultures of Ss, PAO1, or both species with or without 10 µg/mL α-MalE mAbs. After 24 h, bacterial colony-forming units were enumerated (*n* = 6 biological replicates, 10 flies per replicate). Two-way ANOVA with Šίdák’s multiple comparisons test. **B** Quantification of bacterial CFUs per fly after 24-hour colonization with Ss, PAO1, and PAO1*ΔpslA*. Error bars indicate mean ± SD. One-way ANOVA with Tukey’s multiple comparisons test. *p < 0.05, **p < 0.01.

To identify candidate *S. salivarius* proteins that could be involved in *P. aeruginosa*-dependent biofilm promotion, we examined the protein profile of whole-cell lysates of *S. salivarius* and *P. aeruginosa* single and dual cultures (Fig. 4A). Liquid chromatography-tandem mass spectrometry (LC-MS/MS) analysis revealed the overexpression of one ~50 kDa protein that was identified as the *S. salivarius* maltose-binding protein MalE. MalE was overexpressed in dual cultures with *P. aeruginosa*, but not in single *S. salivarius* cultures. To examine whether MalE is involved in *P. aeruginosa*-dependent promotion of *S. salivarius* biofilm formation, we added anti-MalE antibodies to single and dual cultures of *S. salivarius* and *P. aeruginosa*. We found that anti-MalE antibodies significantly inhibited dual biofilm formation in a dose-dependent manner, while growth of single species biofilms was unaffected (Fig. 4B). These findings suggest that MalE is involved in promotion of *S. salivarius* biofilm formation in the presence of *P. aeruginosa*. To determine whether *P. aeruginosa* promotes *S. salivarius* colonization in an in vivo model of co-infection, *Drosophila melanogaster* were co-infected with subcultures of *S. salivarius* and *P. aeruginosa*, and bacterial CFUs were enumerated after 24 h. Colonization was performed with and without 10 µg/mL of α-MalE antibodies to test whether colonization of *S. salivarius* is MalE-dependent. *S. salivarius* colonization significantly increased in the presence of *P. aeruginosa*, while no change in *P. aeruginosa* colonization between groups was observed (Fig. 5A). Consistent with our in vitro data, the addition of 10 µg/mL α-MalE antibodies caused a significant decrease in *S. salivarius* colonization in the dual infection group but had no effect on colonization during single species infections (Fig. 5A). These findings suggest that MalE is required for increased colonization by *S. salivarius* in the presence of *P. aeruginosa*.

To examine whether the promotion of *S. salivarius* colonization is also Psl-dependent in an in vivo model of infection, we infected flies with subcultures of PAO1 and PAO1*ΔpslA* with and without *S. salivarius*, then quantified CFUs per fly after 24 h. The presence of wild-type PAO1 significantly promoted *S. salivarius* colonization, while the presence of PAO1*ΔpslA* marginally, but not significantly, promoted *S. salivarius* colonization (Fig. 5B). Additionally, *S. salivarius* did not promote *P. aeruginosa* colonization. Our results demonstrate that in vivo colonization of *S. salivarius* is both MalE-dependent and Psl-dependent.

### Environmental and clinical non-mucoid isolates of *P. aeruginosa* enhance dual biofilms with *S. salivarius*

**Fig. 6:**
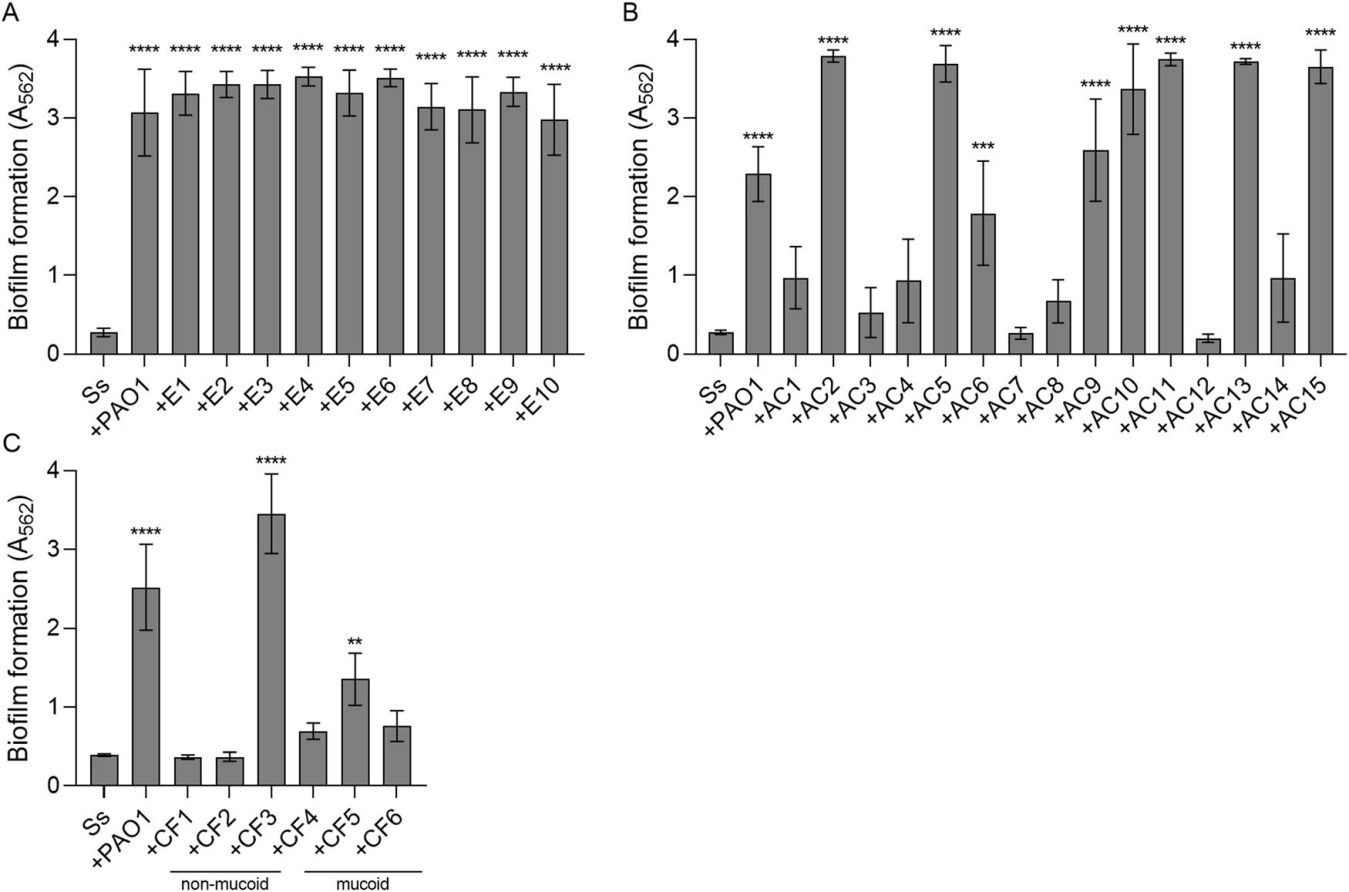
Environmental and clinical isolates of *P. aeruginosa* promote dual biofilm formation. Ss was co-cultured with **A** environmental, **B** non-CF acute, or **C** CF non-mucoid and mucoid isolates of *P. aeruginosa* in TSBYE with 1% sucrose in a 96-well 16-h biofilm model. Biofilms were stained with crystal violet to measure biofilm biomass (*n* = 3 biological, 3 technical). Error bars indicate mean ± SD. One-way ANOVA with Dunnett’s multiple comparisons test (**A** + **B**). One-way ANOVA with Holm-Šίdák’s multiple comparisons test (**C**). **p < 0.01, ***p < 0.001, ****p < 0.0001.

To test whether our enhanced dual biofilm phenotype was consistent with non-lab adapted strains of *P. aeruginosa*, we isolated ten non-mucoid environmental *P. aeruginosa* isolates from local water sources in Birmingham, Alabama. Biofilm formation significantly increased to levels similar to that of PAO1 when all ten isolates were co-cultured with *S. salivarius* (Fig. 6A). *S. salivarius* was also co-cultured with fifteen non-CF clinical *P. aeruginosa* isolates. Out of fifteen isolates, nine produced an enhanced dual biofilm in combination with *S. salivarius* (Fig. 6B). Lastly, three non-mucoid and three mucoid CF clinical *P. aeruginosa* isolates were co-cultured with *S. salivarius* (Fig. 6C). One out of the three non-mucoid CF isolates promoted biofilm formation to a greater degree than PAO1, while one out of the three mucoid isolates significantly promoted dual biofilm formation to a lesser extent than PAO1. Overall, these findings indicate that the ability to promote *S. salivarius* biofilm formation is common among both environmental and clinical isolates of *P. aeruginosa*.

## Discussion

Research in recent years has emphasized that a large percentage of bacteria present within any given environment exists in biofilms, rather than a free-floating, planktonic state. Additionally, bacterial species usually exist within polymicrobial communities rather than mono-species infections [32]. However, few studies have examined the role of polysaccharides in the development of mixed-species biofilms. In this paper, we demonstrate a mechanism by which a commensal bacterium utilizes a polysaccharide from a pathogenic species to promote its own biofilm formation. Our results are significant because this mechanism could potentially change the microbial composition of the CF lung by promoting commensal streptococci colonization.

The importance of studying bacterial infections in the context of the CF lung polymicrobial community is becoming increasingly recognized. A seminal study that reported oral commensal streptococci were associated with improved lung function identified *S. salivarius* as the most abundant oral streptococcus found in the lungs of clinically stable individuals with CF [16]. However, no studies have examined how *S. salivarius* colonizes the CF lung polymicrobial environment and impacts lung function. In this study, we demonstrate *S. salivarius* may incorporate itself into polymicrobial biofilms by using a streptococcal sugar-binding protein, MalE, to interact with the *P. aeruginosa* exopolysaccharide Psl in both in vitro and in vivo fly models of co-infection.

Our findings are significant because interactions between *S. salivarius* and *P. aeruginosa* may have an impact on *P. aeruginosa* virulence and pathogenesis. Although *P. aeruginosa* viability was not reduced in our in vitro and in vivo models of co-infection, *S. salivarius* may potentially interfere with *P. aeruginosa* pathogenesis and impact CF lung function by disrupting Psl availability to *P. aeruginosa*. Psl is a polysaccharide known for its role in resistance against antibiotic treatment and persistence of lung infections [9, 33, 34]. Our findings demonstrate that *S. salivarius* not only uses Psl as a biofilm scaffold, but can also metabolize Psl. This sequestering of Psl by *S. salivarius* could interfere with the ability of *P. aeruginosa* to use Psl as a mechanism for persistence.

We have previously shown that oral commensal streptococci can use cell surface adhesins to bind to the *P. aeruginosa* exopolysaccharide alginate to promote its own biofilm formation [20]. MalE has been shown to be anchored to the cell surface of Gram-positive bacteria, therefore allowing it to interact with extracellular molecules [35]. MalE in Group A *Streptococcus* has a wide range of sugar substrates, which has been implicated in helping *Streptococcus* species adapt to different host environments and support colonization [36]. Additionally, the maltose-binding protein of *Streptococcus mutans* along with many other bacterial species has been shown to also bind and transport sucrose in addition to maltose [37]. The Psl structure contains multiple sugars, including galactose, mannose, rhamnose, and glucose [38], which *S. salivarius* MalE may be utilizing to promote biofilm formation. Our previous findings and current results suggest MalE as a possible candidate protein that facilitates oral commensal biofilm promotion by binding to exopolysaccharides produced by *P. aeruginosa*.

Numerous studies have demonstrated the potential health benefits of *S. salivarius*. In combination with *Streptococcus oralis*, *S. salivarius* has been shown to inhibit biofilm formation by six pathogens that commonly infect the upper respiratory tract, including *Staphylococcus aureus*, *Staphylococcus epidermidis*, *Streptococcus pyogenes*, *Streptococcus pneumoniae*, *Propionibacterium acnes*, and *Moraxella catarrhalis* [39]. Persons with CF infected with *P. aeruginosa* experience respiratory exacerbation episodes characterized by a large inflammatory response associated with increased pro-inflammatory cytokines IL-1, IL-6, IL-8, and TNF-α [40]. *S. salivarius* downregulates the innate immune response to *P. aeruginosa* in infected human epithelial cells [41] and inhibits both the pro-inflammatory NF- κB pathway in vitro and inflammation in an in vivo colitis mouse model [42]. Additionally, *S. salivarius* has been shown to downregulate IL-8 production induced by *P. aeruginosa* in human bronchial epithelial cells [41]. These observations warrant further studying of interactions between *S. salivarius* and *P. aeruginosa* to understand how *S. salivarius* affects the inflammatory host response to *P. aeruginosa* and how, in turn, this affects CF lung tissue damage.

Because *S. salivarius* colonizes the upper respiratory tract early in life and *P. aeruginosa* colonizes individuals with CF more commonly as they age, we would expect *S. salivarius* to be present in the CF lung during the early stages of infection when non-mucoid *P. aeruginosa* is acquired from the environment. All ten of our non-mucoid environmental *P. aeruginosa* isolates were able to produce an enhanced dual biofilm when co-cultured with *S. salivarius*.. The ability of *S. salivarius* to create an enhanced biofilm with environmental strains suggests that *S. salivarius* could be utilizing *P. aeruginosa* Psl from these non-mucoid strains to colonize the lungs during early *P. aeruginosa* infection. Further studies are required to understand how *S. salivarius* colonization impacts early *P. aeruginosa* infection. While only one of the three non-mucoid CF isolates produced an enhanced dual biofilm, the two other isolates displayed colony morphology differing from that of high Psl-producing strains such as PAO1, suggesting that their biofilm matrix is not primarily comprised of Psl. When *S. salivarius* was co-cultured with mucoid CF isolates of *P. aeruginosa*, one isolate produced a significantly enhanced dual biofilm. These results are consistent with previous literature that demonstrates Psl contributes to biofilm formation in some mucoid CF *P. aeruginosa* isolates [43].

In summary, we report a novel mechanism by which *S. salivarius* and *P. aeruginosa* interact within a biofilm and in our in vivo model. Furthermore, we have previously shown that the oral commensal *S. parasanguinis* interacts with the *P. aeruginosa* exopolysaccharide alginate to promote streptococcal biofilm formation [20]. Collectively, these studies illustrate potential mechanisms by which oral commensal streptococci interact with *P. aeruginosa* to alter the composition of the CF airway microbial community. In conclusion, our data suggest a model in which oral streptococci exploit *P. aeruginosa* exopolysaccharides, resulting in enhanced commensal biofilm development. The novel interactions between *S. salivarius* and *P. aeruginosa* revealed in this study could have implications for CF airway microbial community development and warrant further study.

## Materials and methods

### Bacterial strains, culture conditions, and reagents

Strains *S. salivarius* K12, *P. aeruginosa* PAO1, *P. aeruginosa* PAO1*∆pslA*, P. *aeruginosa* PAO1*pslA*^*+*^, *P. aeruginosa* PA14, *P. aeruginosa* FRD1, and *P. aeruginosa* environmental, acute clinical, and CF isolates were used in this study (Table 1). S*. salivarius* was grown on Todd-Hewitt Broth (THB) agar (Becton Dickinson) and cultured statically at 37 °C in 5% CO_2_ in THB. *P. aeruginosa* was grown on Pseudomonas Isolation Agar (PIA; Becton Dickinson) and cultured in Luria broth (LB; Fisher) and incubated while shaking (250 rpm) at 37 °C. PAO1*pslA*^*+*^ and PAO1-GFP were selected for on PIA with 100 μg/mL carbenicillin (Sigma-Aldrich) and were cultured in LB with 100 μg/mL carbenicillin. DH10b (*E. coli*) was cultured while shaking in LB at 37 °C. After transformation, DH10b was cultured in SOC medium (Fisher) while shaking at 37 °C for an hour.

**Table 1:** Bacterial strains and plasmids.

Strain	Characteristics	Reference/source
K12 (*S. salivarius*)	Wildtype	[51]
FRD1 (*P. aeruginosa*)	CF isolate, mucoid	[52]
PAO1 (*P. aeruginosa*)	Wound isolate, non-mucoid	[53]
PAO1*ΔpslA* (*P. aeruginosa*)	In-frame deletion of *pslA*	[27]
PAO1 *pslA*^+^ (*P. aeruginosa*)	Complemented PAO1*ΔpslA* with *pslA* gene	This study
PAO1*ΔmucA* (*P. aeruginosa*)	Deletion of *mucA* (PDO300)	[28]
PA14 (*P. aeruginosa*)	Wildtype	[29]
PAO1-GFP	PAO1 with carb^R^ GFP plasmid	This study
Environmental isolates (*P. aeruginosa*)	E1-E10 non-mucoid, water isolate	This study
AC1 (*P. aeruginosa*)	Non-mucoid, urine isolate	Dr. Bill Benjamin UAB Clinical Microbiology Lab
AC2 (*P. aeruginosa*)	Non-mucoid, wound isolate	Dr. Bill Benjamin UAB Clinical Microbiology Lab
AC3 (*P. aeruginosa*)	Non-mucoid, urine isolate	Dr. Bill Benjamin UAB Clinical Microbiology Lab
AC4 (*P. aeruginosa*)	Non-mucoid, bronchial wash isolate	Dr. Bill Benjamin UAB Clinical Microbiology Lab
AC5 (*P. aeruginosa*)	Non-mucoid, urine isolate	Dr. Bill Benjamin UAB Clinical Microbiology Lab
AC6 (*P. aeruginosa*)	Non-mucoid, blood isolate	Dr. Bill Benjamin UAB Clinical Microbiology Lab
AC7 (*P. aeruginosa*)	Non-mucoid, bronchoalveolar lavage isolate	Dr. Bill Benjamin UAB Clinical Microbiology Lab
AC8 (*P. aeruginosa*)	Non-mucoid, urine isolate	Dr. Bill Benjamin UAB Clinical Microbiology Lab
AC9 (*P. aeruginosa*)	Non-mucoid	Dr. Bill Benjamin UAB Clinical Microbiology Lab
AC10 (*P. aeruginosa*)	Non-mucoid, tracheal aspiration isolate	Dr. Bill Benjamin UAB Clinical Microbiology Lab
AC11 (*P. aeruginosa*)	Non-mucoid, urine isolate	Dr. Bill Benjamin UAB Clinical Microbiology Lab
AC12 (*P. aeruginosa*)	Non-mucoid, urine isolate	Dr. Bill Benjamin UAB Clinical Microbiology Lab
AC13 (*P. aeruginosa*)	Non-mucoid, urine isolate	Dr. Bill Benjamin UAB Clinical Microbiology Lab
AC14 (*P. aeruginosa*)	Non-mucoid, nasal isolate	Dr. Bill Benjamin UAB Clinical Microbiology Lab
AC15 (*P. aeruginosa*)	Non-mucoid, maxillary sinus isolate	Dr. Bill Benjamin UAB Clinical Microbiology Lab
CF clinical isolates (*P. aeruginosa*)	CF1-CF3 non-mucoid, CF4-CF6 mucoid	Dr. Susan Birket UAB CF Center
DH10b (*E. coli*)	Host strain for cloning	Thermo Fisher
pBKSNS1	pBluescript K(+) ligated to *pslA* gene	This study

### Biofilm formation assays

Overnight cultures of *S. salivarius* and *P. aeruginosa* were sub-cultured in THB and LB, respectively, and grown to exponential phase (OD_600_ 0.5–0.8). Sub-cultures were then inoculated into Tryptic Soy Broth (MP Biomedicals) with 0.5% yeast extract (Fisher) (TSBYE) containing 1% sucrose at a dilution of 1:1000 for *S. salivarius* and 1:100 for *P. aeruginosa*. The two strains were inoculated either separately for the single species biofilm or together for the dual species biofilm assays. 200 μL of each sample was added to a 96-well plate (Nunc) in triplicate and incubated statically at 37 °C in 5% CO_2_ for 16 h. The biofilms were then stained with 0.1% crystal violet and dissolved in 30% acetic acid [44]. Absorbance was measured at 562 nm to quantify biofilm biomass using the Synergy HTX Multi-Mode Microplate Reader (BioTek).

### Quantification of *P. aeruginosa* and *S. salivarius* in co-cultures

Cultures were grown in either TSBYE with 1% sucrose, or synthetic cystic fibrosis sputum (SCFM2). SCFM2 was made as previously described [23]. To quantify colony forming units of each species, serial dilutions in TSBYE of planktonic samples from a 6-h six-well biofilm assay were plated on THB agar square plates (Fisher) using the track dilution method [45]. Remaining planktonic cells were aspirated off, and adherent biofilm cells were then washed two times with phosphate-buffered saline (PBS), scraped and resuspended in 3 mL of TSBYE. The resulting suspension was serially diluted and plated.

### Construction of the PAO1 *pslA*^*+*^ complemented strain

The *pslA* gene was cloned by PCR amplifying ~500 bp upstream and downstream of the coding region from the wildtype PAO1 strain using primer sequences described (Table 2). The PCR product was cloned into the EcoRI and BamHI sites of the pBluescript K(+) shuttle vector (Addgene). The resulting plasmid, referred to as pBKSNS1, was converted to a mobilizable plasmid by incorporation of a m*oriT* into the HindIII site [46] and transformed into competent *E. coli* strain DH10b using a standard transformation method [47]. The pBKSNS1 plasmid was introduced into PAO1*ΔpslA* through triparental mating [46]. Plasmid conjugation events were selected for using PIA with 100 μg/mL carbenicillin.

**Table 2:** Primer sequences.

Gene	Forward or Reverse	Sequence (5′−3′)	Amplicon size (bp)
*pslA*	Forward	GGATTGGCGGCGTCAGATTT	2207
	Reverse	TCGATATAGCCGAAGCCGGT	
*pslA* (qPCR)	Forward	CATGCACCTGGCCGAATA	109
	Reverse	CGGCAGCGAGTTGTAGTT	
16S rRNA	Forward	GCTGGACTATCGCCGCTG	150
16S rRNA	Reverse	ATCTCGTAACCGGTGAAGGTG	

### Psl purification

Purification was performed on PAO1*pslA*^*+*^ cultures grown overnight at 37 °C in six-well plates in TSBYE supplemented with 1% sucrose and 100 µg/mL carbenicillin. Cultures were pooled together, diluted 1:1 with 0.9% NaCl, and agitated with 0.01 M EDTA by centrifuging at 200 rpm for 30 min at 4 °C to detach cell-associated Psl. Cultures were then centrifuged at 10,000 *g* for 15 min at 4 °C to remove bacterial cells. The resulting supernatant was filtered with a 0.22 μm vacuum filter to remove excess cell debris. Exopolysaccharide was then precipitated with 1:1 volume of cold 100% ethanol for 1 h at −80 °C, and resulting precipitate was centrifuged at 15,000 *g* for 15 min at 4 °C. The pellet was resuspended in PBS containing 1 mM CaCl_2_ and 10 mM MgCl_2_ and was subsequently treated with DNase I (100 µg/mL), RNase A (100 µg/mL), and Proteinase K (100 µg/mL) for 2 h at 37 °C, then lyophilized [48].

### *pslA* quantification

Biofilms were cultured in TSBYE supplemented with 1% sucrose in six-well plates for 6 h in 5% CO_2_. Biofilms were washed with PBS, and adherent bacteria was resuspended. RNA was isolated using the Direct-zol RNA Mini Prep kit (Zymo Research). cDNA conversion was performed with the iScript cDNA Synthesis kit (Bio-Rad), on the CFX96 Real-Time PCR System (Bio-Rad). *P. aeruginosa* 16S rRNA was used to quantify total RNA present in samples. Primers specific to *P. aeruginosa pslA* and 16S rRNA are listed in Table 2. The delta-delta CT method was used to calculate fold change of gene expression.

### SDS-PAGE and mass spectrometry protein analysis

*S. salivarius* and *P. aeruginosa* were cultured planktonically in TSBYE medium at 37 °C in single and dual cultures until OD_600_ ~1.8 was reached. Cultures were spun down, resuspended in tris-buffered saline, and lysed using the Bead Blaster 24 (Benchmark Scientific). Cell debris was centrifuged, and the resulting supernatant was mixed with 6× Laemmli buffer and ran on an SDS Page gel. Overexpressed bands were excised from the gels and digested with trypsin. The digested peptide fragments were analyzed for protein identification by LC-MS/MS as described previously [49] and sent to the UAB Mass Spectrometry Core for identification. We repeated the SDS-PAGE experiment three times, the only reproducible change was this ~50 kDa band. We also excised the bands from three biologic replicates and analyzed by mass spectrometry.

### Immunofluorescence and confocal laser scanning microscopy

Bacterial strains were grown in either TSBYE supplemented with 1% sucrose or in SCFM2 in a sterile eight-well treated μSlide (Ibidi). Samples were incubated at 37 °C under 5% CO_2_ for 16 h. Biofilm samples were gently washed twice with PBS to eliminate planktonic bacteria and then incubated in PBS with 1% bovine serum albumin (BSA) for 10 min. Samples were then stained for 10 min with hexidium iodide (Thermo Fisher) at a 1:1000 dilution and a FITC-conjugated α-Psl antibody (Creative Biolabs) at a 1:100 dilution in PBS with 1% BSA. The samples were washed with PBS once more and then analyzed for fluorescence using the Nikon A1R Confocal Laser Scanning Microscope (Nikon Instruments Inc.) at the University of Alabama at Birmingham High Resolution Imaging Facility.

### Drosophila melanogaster colonization assay

*Drosophila melanogaster* flies were maintained on Jazz-mix Drosophila food (Fisher). Three to seven-day-old flies were treated with antibiotics for 3 days (50 µg/mL vancomycin, 50 µg/mL erythromycin, and 50 µg/mL ampicillin). Flies were separated into vials in groups of ten and subsequently starved for 3 h prior to infection. *S. salivarius* and *P. aeruginosa* cultures were grown to A_600_ 2.0. For single species infection groups, 1.5 mL of the respective culture was centrifuged for 6 min at 6000 *g* and resuspended in 100 µl of 5% sucrose. For dual infection groups, 0.75 mL of each culture were combined, centrifuged, and resuspended in 5% sucrose. Resuspended cultures were then pipetted onto sterile 21 mm filter disks (Whatman) and placed into plastic vials containing 5 mL of 5% sucrose agar. After 24 h of infection, flies were briefly washed in 70% ethanol to remove outside contaminants and then washed with sterile PBS. Flies were crushed and resuspended in 500 µl PBS. The resulting homogenate was then serially diluted and plated on THB agar plates using the track dilution method to quantify bacterial colony-forming units inside flies.

### Statistical analysis

All graphs represent sample means ± SD. The Shapiro-Wilk normality test was used to determine distribution of datasets. Statistical analysis of normally distributed data was performed using either Student’s *t* test or one-way ANOVA with Tukey’s multiple comparisons test. For experiments with two or more factors (for example, absorbance and treatment), a two-way ANOVA was used with either Tukey’s or Šίdák’s multiple comparisons test. Tests were performed using Graphpad Prism version 9 for Windows, La Jolla California USA, www.graphpad.com. Data were considered statistically significant if *p* < 0.05.

## Supplementary information

Supplemental Materials
