# Supplementary material for: Pseudomonas aeruginosa polysaccharide Psl supports airway microbial community development
Source: ISME J. 2022 Mar 25;16(7):1730–9. doi: 10.1038/s41396-022-01221-y (PMC9213427; doi:10.1038/s41396-022-01221-y)
Supplement: Supplementary file 1 — Supplemental Materials [file 41396_2022_1221_MOESM1_ESM.docx]

**Figure S1: Planktonic CFUs of *S. salivarius* and *P. aeruginosa* in TSBYE and SCFM2**


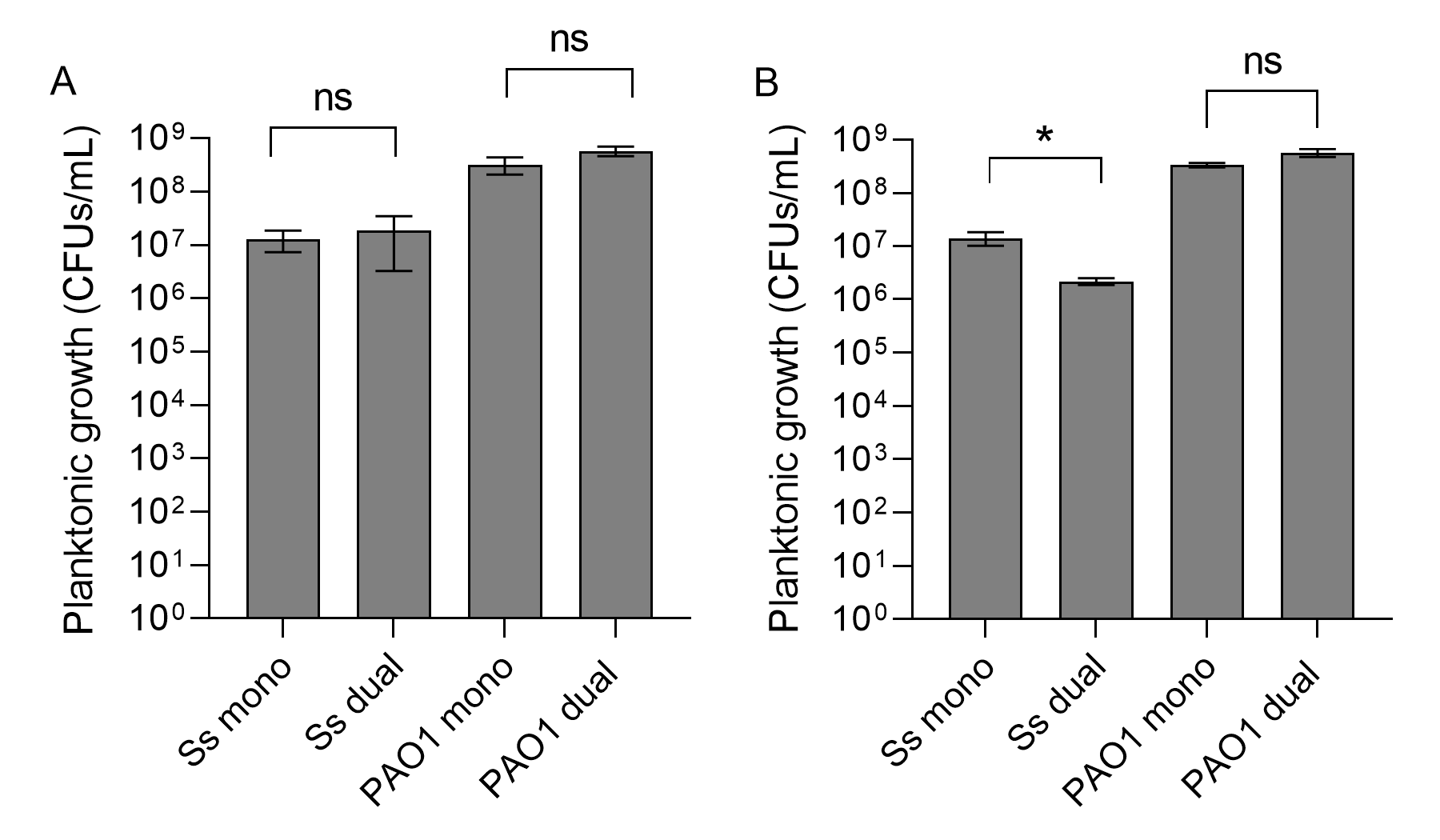


Ss and PAO1 were cultured individually and dually in both **(A)** TSBYE medium with 1% sucrose and **(B)** SCFM2 in 6-well 6-hour biofilm model. Planktonic samples were collected, serially diluted, and plated on THB agar plates. **p* < 0.05

**Figure S2: Quantification of dead cells in single and dual biofilms**


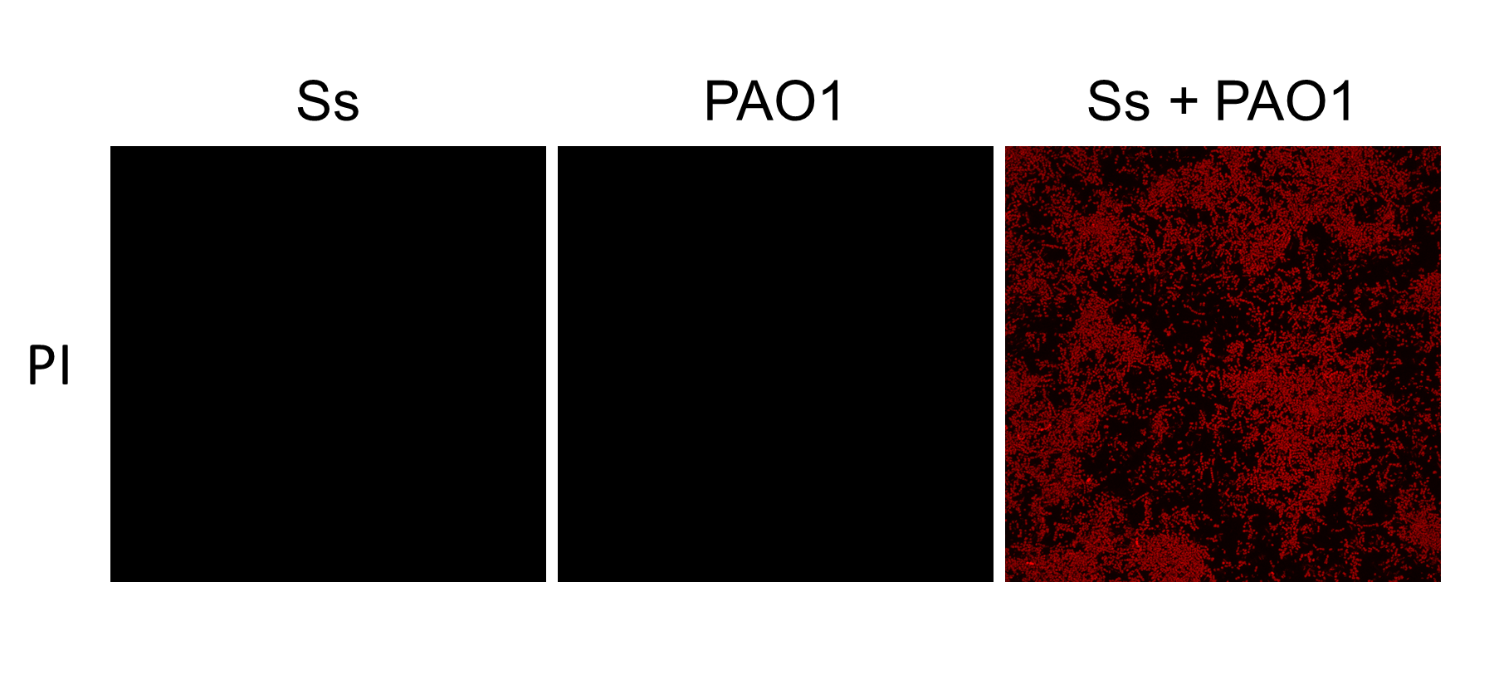


Fluorescence microscopy images at 60x magnification of 16-hour single and dual species biofilms of Ss and PAO1 cultured in TSBYE supplemented with 1% sucrose. All samples were stained with propidium iodide. Scale bar: 20 μm.

**Figure S3:** **Planktonic and biofilm CFUs of Ss, PAO1, PAO1*ΔpslA*, PAO1 *pslA*^+^ in TSBYE**


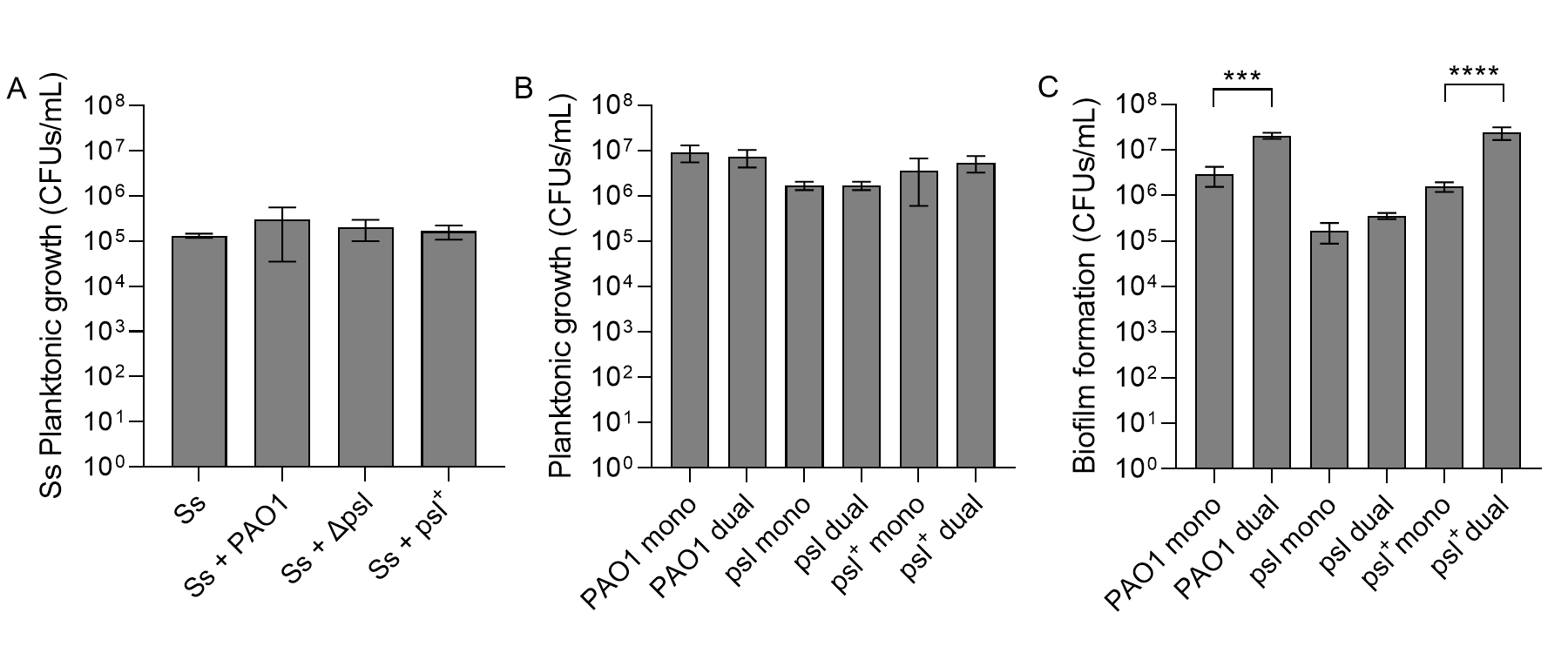


**(A)** Ss planktonic CFUs in the presence or absence of PAO1, PAO1*ΔpslA*, or PAO1 *pslA*^+^ (n=3 biological, 3 technical). One-way ANOVA with Dunnett’s multiple comparisons test. **(B)** PAO1, PAO1*ΔpslA*, or PAO1 *pslA*^+^ planktonic CFUs in the presence of absence of Ss. One-way ANOVA with Šίdák’s multiple comparisons test. **(C)** PAO1, PAO1*ΔpslA*, or PAO1 *pslA*^+^ biofilm CFUs in the presence of absence of Ss. One-way ANOVA with Šίdák’s multiple comparisons test. ****p* < 0.001, *****p* < 0.0001.

**Figure S4: Planktonic and biofilm CFUs of Ss, PAO1, PAO1*ΔpslA*, PAO1 *pslA*^+^ in SCFM2**


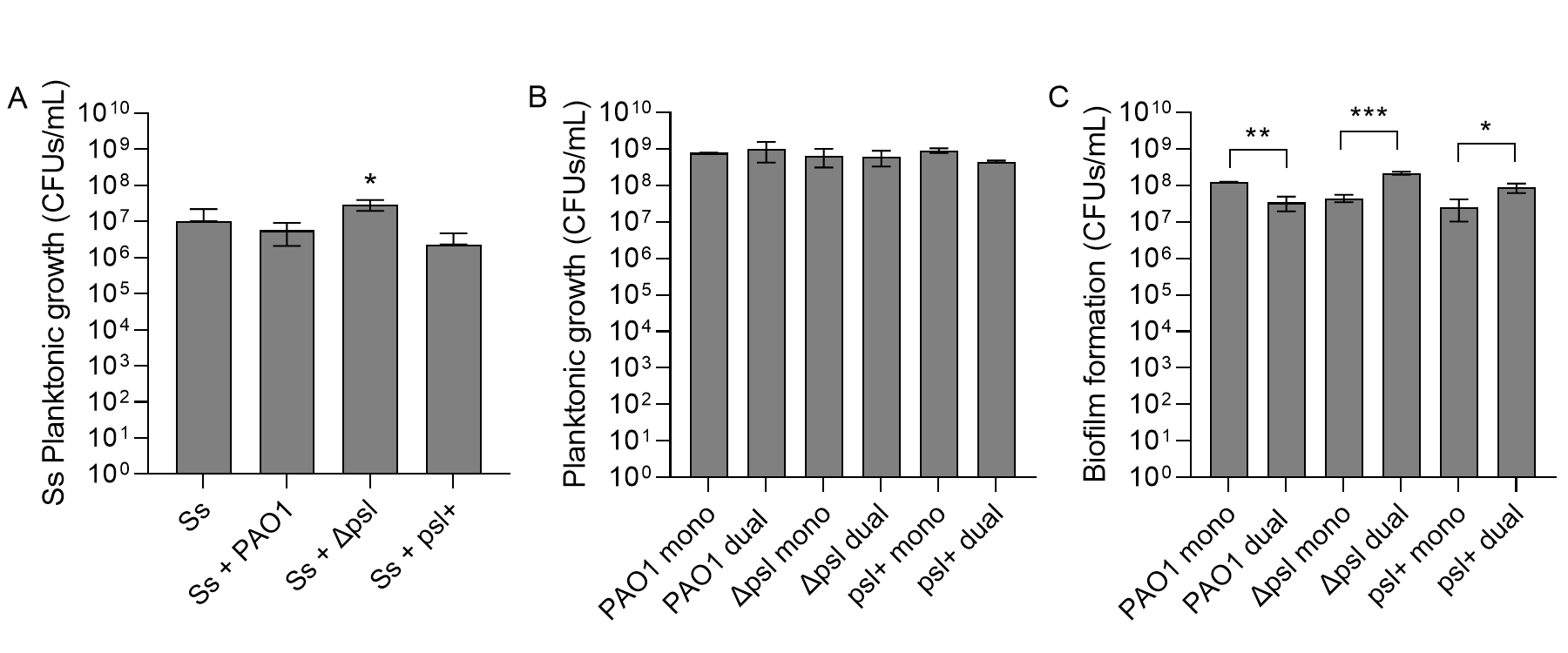


**(A)** Ss planktonic CFUs in the presence or absence of PAO1, PAO1*ΔpslA*, or PAO1 *pslA*^+^ (n=3 biological, 3 technical). One-way ANOVA with Dunnett’s multiple comparisons test**. (B)** PAO1, PAO1*ΔpslA*, or PAO1 *pslA*^+^ planktonic CFUs in the presence of absence of *Ss*. One-way ANOVA with Šίdák’s multiple comparisons test. **(C)** PAO1, PAO1*ΔpslA*, or PAO1 *pslA*^+^ biofilm CFUs in the presence of absence of Ss. One-way ANOVA with Šίdák’s multiple comparisons test. **p* < 0.05, ***p* < 0.01, ****p* < 0.001

**Figure S5: Sixteen-hour growth curves of *S. salivarius* and *P. aeruginosa***


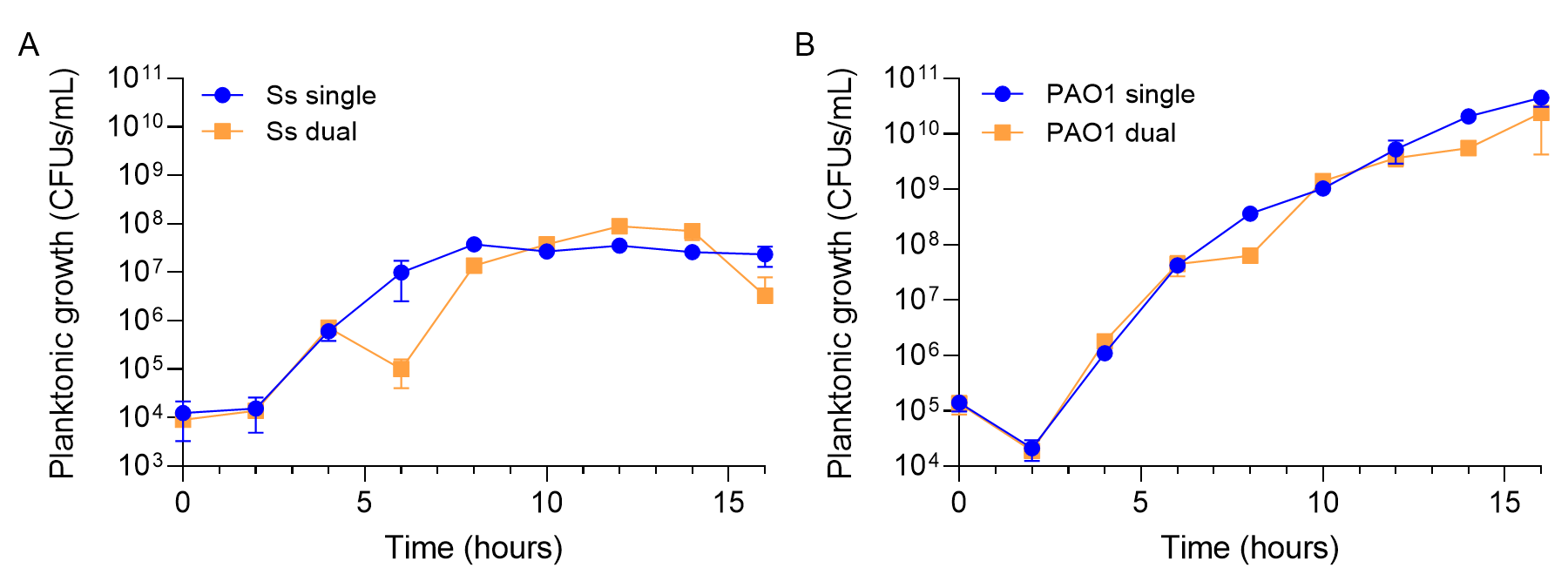


**(A)** Ss was cultured in the absence and presence of PAO1 and **(B)** PAO1 was cultured in the absence and presence of Ss in TSBYE with 1% sucrose in 6-well plates at 37 ̊C in 5% CO_2_. Samples were collected, diluted, and plated on THB agar every two hours for a total of eight hours.

**Figure S6: *P. aeruginosa* strains that do not produce Psl fail to create an enhanced dual biofilm with *S. salivarius***


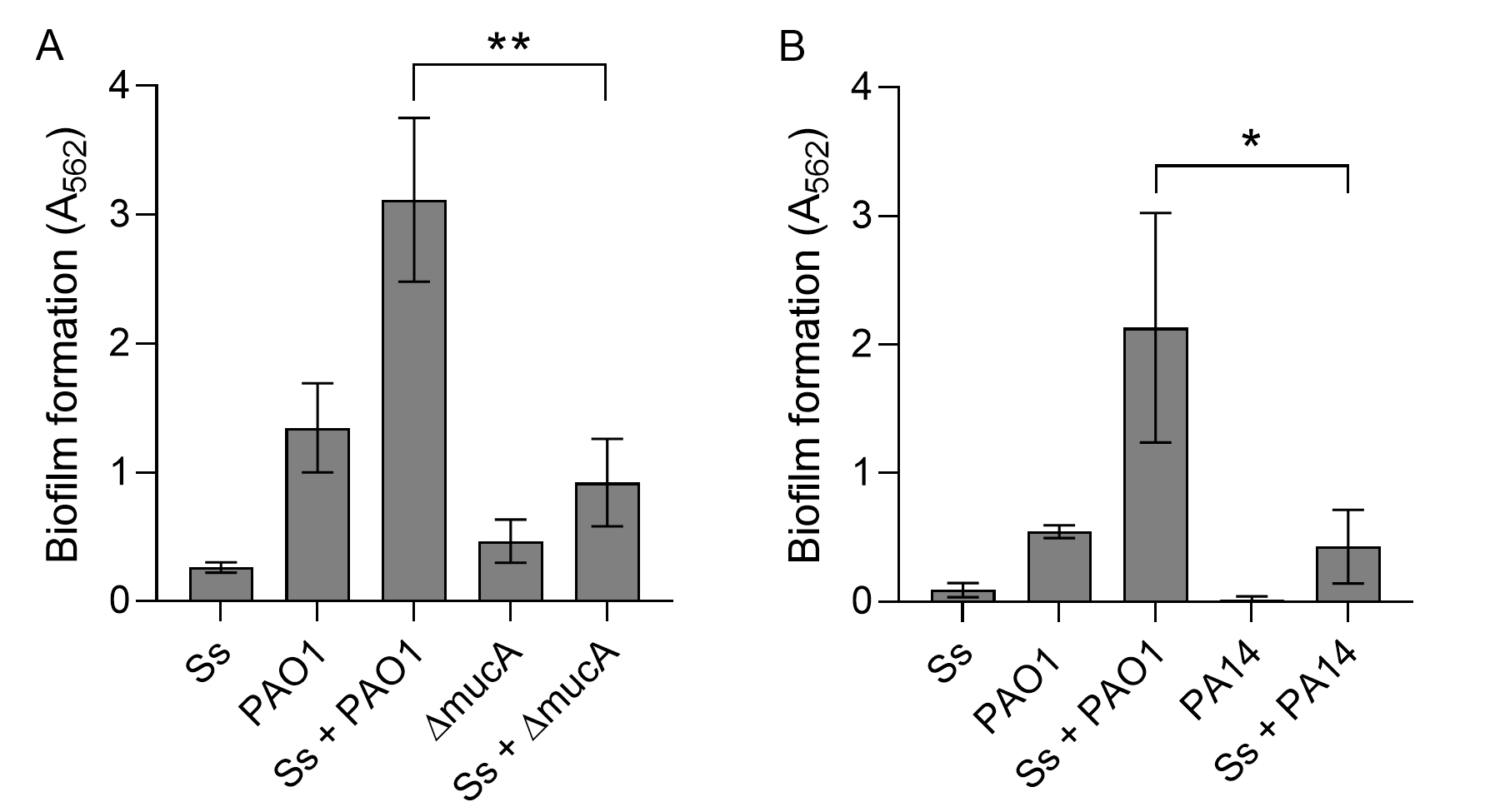


Ss was co-cultured with **(A)** PAO1∆*mucA* and **(B)** PA14 in TSBYE with 1% sucrose in a 96-well 16-hour biofilm model and stained with crystal violet to measure biofilm biomass. Error bars indicate mean ± SD. Student’s t-test. **p* < 0.05, ***p* < 0.01

**Figure S7: Metabolism of Psl by *S. salivarius***


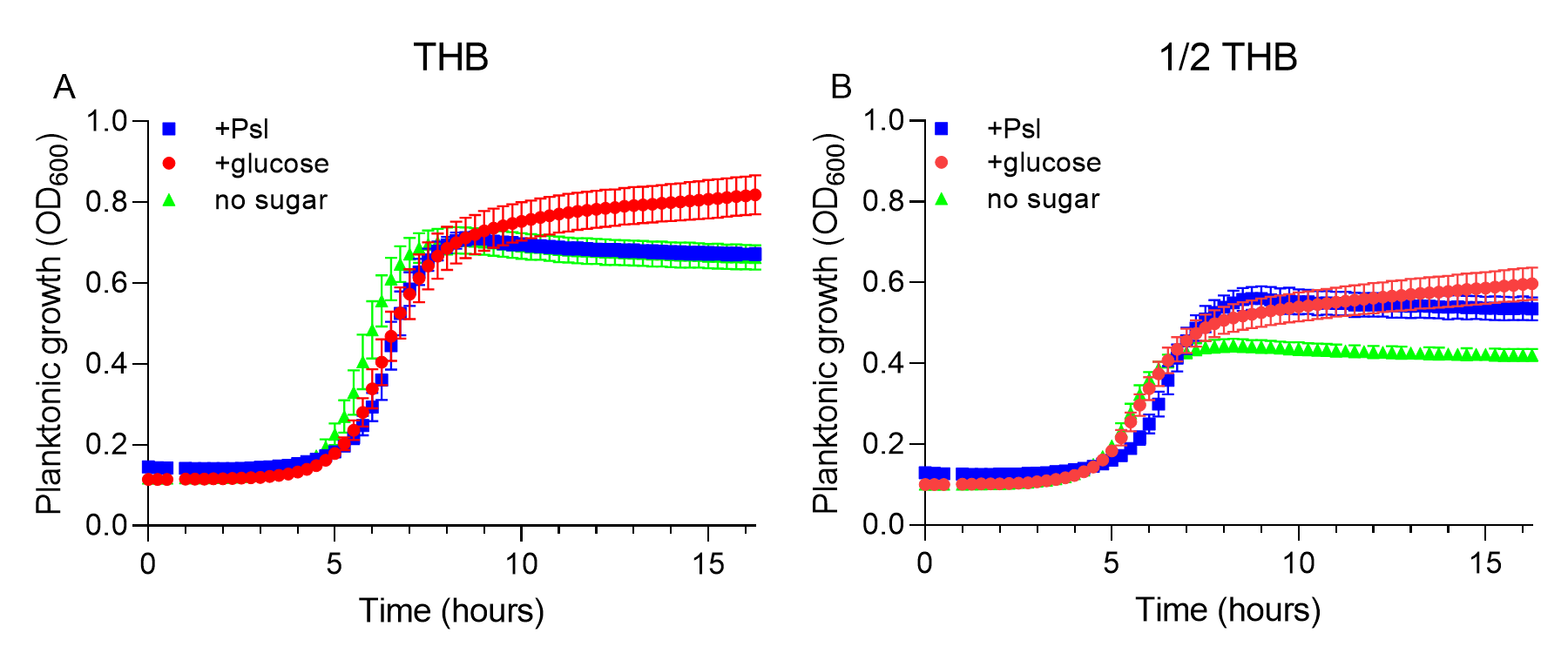


Ss was cultured in **(A)** Todd-Hewitt Broth (THB) or **(B)** 1:1 diluted THB in the presence of 10mg/mL glucose, 10mg/mL Psl, or no sugar. OD_600_ was measured every 15 minutes for 16 hours to quantify growth.
